# Diagnosis of Cholelithiasis and Cholecystitis, with Remarks on Medical Treatment1Read at the 36th annual meeting of the Medical Association of Central New York, held at Auburn, September 22, 1903.

**Published:** 1904-04

**Authors:** Charles G. Stockton

**Affiliations:** Buffalo, N. Y.; Professor of Medicine, University of Buffalo


### BUFFALO MEDICAL JOURNAL.

VOL. XLIII.—LIX. APRIL, 1904. No. 9.

### ORIGINAL COMMUNICATIONS.

### Diagnosis of Cholelithiasis and Cholecystitis, with Remarks on Medical Treatment.[1]

By CHARLES G. STOCKTON, M. D., Buffalo, N. Y.
Professor of Medicine, University of Buffalo.

IT IS generally held that an important if not necessary feature in the etiology of cholelithiasis, is to be found in a disturbance of the epithelial lining of the gallbladder. Given a perversion of the nutrition of, and exudation from, the lining of this viscus and the formation of biliary calculi may be expected. A mass of evidence has accumulated which goes to show that, in the majority of instances, the disturbance in the lining of the gallbladder is most commonly brought about through the entrance of infection into the organ and the setting up of either a mild or severe cholecystitis. Statistics show that there are certain predisposing factors of importance, among which are age, sex, indolence, gluttony and constipation. The disease is most often observed in stout, constipated, inactive women of middle age or older.

So closely associated is cholelithiasis with cholecystitis, both in the matter of etiology and clinical history, that the diagnostician must necessarily consider both processes, and it is not always easy to separate the phenomena depending upon the one from those depending upon the other. In acute inflammation of the gallbladder occurring in typhoid fever with the presence of the pure culture of Eberth's bacillus, we have, perhaps, the most classic instance of uncomplicated cholecystitis; and yet, in operating for the relief of this condition, newly formed and relatively

1. Read at the 36th annual meeting of the Medical Association of Central New York, held at Auburn, September 22, 1903.

soft gallstones containing living typhoid bacilli are sometimes found. In chronic cases following this infection calculi are almost invariably present. Naturally, the personal history of the patient as to age, activity, habits of life and previous infections, has some bearing in the diagnosis.

### ACUTE CHOLECYSTITIS.

This condition usually gives rise to distention of the gallbladder, with either serous or purulent fluid. This accumulation of fluid depends upon the closure of the cystic duct as a result of inflammatory swelling, and the pouring out of an inflammatory exudate more or less rich in cellular elements of the blood and epithelium of the gallbladder, depending upon the activity of the process. The affection is accompanied by local and constitutional disturbances. Locally, there is increased tension, and the soft parts in the vicinity acquire an increased rigidity. The gallbladder may protrude below the liver, and may be palpated as a rounded mass, continuous with the right lobe of the liver, which, later, is usually somewhat enlarged. Not infrequently there is an accompanying localised angiocholitis, in which case there is likely to be also a more or less active and extensive pericholecystitis with adhesions.

The region is tender, painful, sometimes showing prominence upon inspection, and it is to be noted that the process occurs along the lower rather than upper border of the liver, affecting the right rather than the left lobe. The pain is sometimes very severe, sometimes only moderate, but is rarely altogether absent. There is usually continuous suffering, augmented by paroxysms of severe pain like the pain of biliary colic. The disease is accompanied by fever, usually with rigors or even severe chills, occasionally by marked remissions and sweating; as a rule, there is vomiting, often constipation with moderate tympanitis, at times accompanied by jaundice with its attending symptoms. A leucocytosis is to be expected, and I have known the white cells to rise to 25,000 or 30,000 even when pus was not present. The patient is sometimes gravely ill, and the destructive processes are so active that drainage by surgical intervention is necessary to save life. In other cases the inflammation subsides, to recur, perhaps, repeatedly. Sometimes during the periods of improvement numerous, small, soft biliary calculi are passed by the rectum.

From this description it will be seen that it is impracticable to differentiate between acute cholecystitis with and without cholelithiasis, and it is not always possible to distinguish between a purulent and a nonpurulent case. Sometimes the obstruction of the cystic duct is relatively permanent, in which case there may result either hydrops of the gallbladder or empyema of the gall-bladder. The development of either of these may be recognised from the large, elastic, rounded tumor occurring in the abdomen below the right lobe of the liver. In the absence of acute inflammation one would not expect to find a leucocytosis except when the contained fluid is purulent in character; but when the active inflammation has subsided, the leucocyte count cannot be depended upon as a positive guide, for then even in purulent cases, the count is sometimes not very high.

### MILD OR SUBACUTE CHOLECYSTITIS.

While this condition is less frequently described by authors it must be one which often occurs and which undoubtedly, is often overlooked because the signs and symptoms are inconspicuous. One has the opportunity of studying this condition in cases that have begun as acute processes, but in which the inflammation has subsided. Here the severe pain and tenderness, the fever, digestive derangement and other symptoms markedly subside and some of them entirely disappear, but enough remain to hold the attention when once directed to the diseased part. The liver sometimes returns to its normal size, but usually is slightly enlarged, especially in that part of the right lobe lying immediately over the gallbladder. This portion of the liver sometimes still further enlarges and extends downward in "a tongue-like projection," the so-called process of Riegel. It generally may be found when gallstones are present, and when the local irritation is considerable and protracted.

Under these conditions the gallbladder itself is likely to shrink from the inflammatory thickening and subsequent contraction of its walls, and therefore is no longer palpable. Still one finds cases in which the gallbladder remains large, extending below the liver, even when the right lobe is increased in bulk. In such cases the walls of the gallbladder are usually thin, and the tumor may appear elastic upon palpation. A gallbladder which has once been inflamed is often adherent to the liver, the omentum, or the intestine. These persistent adhesions may give rise to a special train of symptoms. When the adhesions extend to the abdominal wall there may be a sense of tension or even pain when the patient assumes the upright position. When attached to the pylorus they may give rise to important symptoms which are most marked toward the completion of the gastric digestion, when the pylorus is most active, and there may result symptoms of food stagnation with uneasiness, pain and sense of distention, eructation of gas, water brash, ending with nausea and vomiting.

Symptoms resembling the above-named sometimes result from adhesions to the duodenum. In some instances of adhesions the symptoms are less localised, and there is found to be a vague disturbance of the entire digestive tract with accompanying auto-intoxication, constipation, diarrhea, or other digestive derangements.

The diagnosis of these adhesions is not always easily made, especially when no evidence of disease of the gallbladder can be determined by physical examination. A correct diagnosis is sometimes to be made by exclusion, and bearing in mind the fact that the symptoms develop subsequent to an attack of recognised cholecystitis, an intercurrent attack of biliary colic, when it occurs, will assist one materially in reaching a correct conclusion. When chronic irritation, varied by periods of mild inflammation, of the gallbladder exists, it may produce other symptoms that assist in the diagnosis. In some cases there is a sub-icteric appearance of the skin and conjunctivæ, often transient, which betrays the existence of occasional attacks of mild angiocholitis. Some patients are predisposed to attacks of distinct jaundice. At such time there is a sensation of weight, or even pain, in the right hypochondrium or epigastrium, tenderness at the juncture of the ninth rib and cartilage, and sometimes at the point of the twelfth right intercostal nerve, an inch from the spine. This, the so-called Boas sign, is only occasionally present.

Some patients suffer from a continued autointoxication, with bad breath and disagreeable taste, slight elevation of temperature, and characteristic urinary disturbance. Other patients go for years suffering more or less constantly in this way without having attacks of hepatic colic and, therefore, without a diagnosis being made. There are still other instances of mild cholecystitis with cholelithiasis that are even more difficult of recognition. Again, some of these patients complain of gastric symptoms, which are sometimes extremely vague and not to be differentiated from irritative gastric disturbances, such as arise so frequently from deranged nervous systems, reflex irritation, and the like. Occasionally there is complaint of severe attacks of gastralgia, not at all resembling ordinary biliary colic, yet of marked severity, and often regarded as a pure neuralgia.

Still, again, patients suffer from irregularity of the bowels, which condition disappears when relieved from the biliary disease. In fact the disease varies in severity of symptoms, ranging from cases having severe attacks of gallstone colic that accompany acute cholecystitis, to those cases in which the symptoms fail to attract attention, but in which gallstones are formed in a diseased gallbladder, and are only discovered accidently or after death. In a large proportion of instances in which gallstones are found post-mortem, there is no history to indicate that the patient had suspected the presence of cholelithiasis.

### BILIARY OR HEPATIC COLIC.

When symptoms of biliary or hepatic colic occur at irregular intervals which sometimes can be traced to a preceding typhoid infection, or other known cause, it is the form of cholelithiasis most commonly met with. These attacks vary so greatly in their accompanying symptoms that it would be a difficult and tedious matter to describe them all. These differences in symptoms depend in part upon the location of the stone, in part upon the condition of the gallbladder and surrounding structures, and in part upon the resisting power of the individual. The diagnosis is sometimes obscured by the presence of carcinoma in the vicinity, and a tumor in the head of the pancreas may so press upon the ductus choledochus, with jaundice, digestive disturbances, and other symptoms that it may be mistaken for cholelithiasis. As a rule, under these circumstances, the gallbladder is distended and palpable. This group of circumstances was formerly confused with stone in the common duct accompanied by jaundice.

It has been pointed out by Courvoisier, however, that when stone in the common duct leads to obstruction, the gallbladder is not often distended, but on the other hand contracted. There is usually, but not uniformly, pain complained of when the stone is thus located. Jaundice is not invariable for the reason that obstruction is not usually continuous; the stone moves, or the inflammatory edema subsides, so that there are periods in which bile coloring matter appears in the stools. The jaundice fades to deepen with the reappearance of the obstruction. When the stone engages at the juncture of the cystic and hepatic duct and there remains, the resulting symptoms cannot always be distinguished from those following the presence of stone at the outlet of the choledochus. In the former condition the jaundice is apt to be persistent and the evidence of tension in the gallbladder is relatively continuous. Often there is constant distress in the hypochondrium, varied by pain, temperature, vomiting and other symptoms of gallbladder irritation. These cases are often very severe.

Biliary colic frequently occurs when the calculus has not invaded the ducts. Occasionally, the calculus engages in the opening of the cystic duct and through irritation, thus set up produces marked spasm of the viscus with intense pain. Colic attacks are probably not always produced from such behavior of the calculus, for the reason that sometimes we observe these attacks in cases in which the calculus is quite too large to engage in the duct. In these instances the pain is probably produced by the irritation offered by the stone to an already diseased and irritated gallbladder thereby producing swelling, occlusion of the duct and spasm of the viscus.

The sufferers from gallstone disease apparently have attacks of biliary colic induced by disturbances of digestion, over-eating and the development of a condition of toxemia usually described by the term lithemia. It would seem to me that under these circumstances the tissues of the gallbladder become more susceptible to irritation, just as patients who are victims of chronic appendicitis, tonsillitis or bronchitis may experience a reignition of the smouldering trouble through the development of the nutritive disorders just referred to. This is a point of some importance in diagnosis because the increased acidity of the urine which occurs in the cases just referred to, might mislead the observer into supposing that the pain came from irritation to the right kidney or ureter, or the group of symptoms which are sometimes experienced in those suffering from nephroptosis and which passes under the name of Dietl's crisis. There should be no great difficulty in deciding between these conditions, but I have known a difference of opinion to arise between physicians regarding them.

When in the course of cholelithiasis a severe and continuous pain develops with swelling and rigidity in the region of the gallbladder, together with great tenderness, fever, vomiting and retching, and usually considerable tympanitis, we have reason to suspect that the patient is suffering from pericholecystitis. Some recent observers go as far as to attribute all cases of biliary colic to localised peritonitis. We should not forget that in the present instance at least there is a localised peritonitis, and that it may extend sufficiently to produce the gravest results. The danger of perforation should always occur to one under these circumstances. While in fortunate cases, as the result of ulceration, a passage may be formed between the gallbladder and intestines thus leading to drainage of the gallbladder, possibly the escape of calculi, sometimes of very large size, and even ultimate cure of the case, it too often happens that a perforation into the peritoneal cavity results from the process and following which a rapid septic peritonitis is to be expected. Many of the fatal cases of cholelithiasis follow this course.

MEDICAL TREATMENT OF CHOLECYSTITIS AND CHOLELITHIASIS.

Treatment seems entitled to brief consideration in this place. While it is not only useless but reprehensible to delay the assistance which surgical intervention alone can bring to many cases, we are not yet in a position where it is proper to demand a cholecystotomy as soon as a diagnosis of gallstone has been made. The majority of cases can be greatly benefited by appropriate medical treatment, and many will completely recover by medical treatment alone. That which seems to me of the most importance in this medical treatment is the adoption of a regimen that will prevent the appearance of a lithemic condition, a state which experience teaches is likely to induce an irritable gallbladder and precipitate a seizure of biliary colic. In other words, the patient should not be allowed to become constipated, to have scanty and acid urine, to develop gastric derangement in which a coated tongue, disagreeable taste, flatulence and epigastric distress are familiar symptoms. Such patients should be made to eat simply, drink freely of water, pay special attention to the activity of the skin, exercise moderately and progressively, avoid excesses of all kinds, and assist in the elimination of waste matters by a course of alkaline saline waters, with the judicious taking of some preparation of salicylic acid. The plan selected must depend somewhat upon the resisting power of the patient, the condition of the blood, and the viscera; and the physician who understands how to avoid the recurrence of bronchitis in one subject to uricacidemia will be likely to understand the best method of preventing an irritable gallbladder. When the attack appears the spasm must be relieved, and for this perhaps morphine and atropine will be necessary.

When the pain is not intense it may be relieved and the attack aborted by the free administration of salol, sodium salicylate, aspirin, antipyrin, and similar preparations. The patient must be kept absolutely quiet, the alimentary tract emptied carefully without producing too much intestinal peristalsis and food must be denied. The drinking of water and sweating the patient are useful measures, and in a few instances the administration of pilocarpin has for more than one reason seemed to be of value.

As to the use of olive oil in relieving the symptoms of irritable gallbladder we have a good deal of recorded experience, and for the most part it is favorable. Some advise very large, others small doses. The plan which seems to be most serviceable is to administer a teaspoonful of oil every two hours for several consecutive days. In case the oil leads to derangement of gastric digestion it should be abandoned.

We shall undoubtedly do best in our treatment of these cases if we devote more time and thought to the conditions predisposing to the attacks.

436 Franklin Street.

DISCUSSION ON THE PAPERS OF DRS. STOCKTON AND CURTIS.[1]

Dr. W. S. Cheeseman, Auburn.—I would like to ask Dr. Curtis whether he has formulated any rules in relation to the removal of the gallbladder?

Dr. H. L. Elsner, Syracuse.—It seems to me these valuable contributions, on a subject which is interesting all of us, both physicians and surgeons, merit discussion. A great many points have been brought out by both the readers of the papers, and they need to be accentuated. For example, I agree with the statement which Dr. Stockton made, that in those cases of persistent cholecystitis, as a rule, with, but sometimes without gallstone formation, there is the characteristic enlargement of the liver which he describes, the tongue-shaped piece of liver which comes down and covers the gallbladder, which can be palpated before operation, and which often misleads the diagnostician, because he imagines he is dealing with an enlarged gallbladder, when in truth at the time of the operation we often find the gallbladder hidden beneath this piece of enlarged liver.

Then there is another point in connection with this, and perhaps Dr. Stockton's experience may coincide with mine—namely, that there is a change of color in the liver. I have noticed in these cases a leaden hue which we do not find in any other disease of the liver, and that leaden hue is associated with more or less localised cicatricial change. These livers after the gallstones have been removed and the gallbladder is drained return to the normal size, and the only trouble which we ultimately have if the gallbladder has been removed, or if all of the gallstones have been removed, is possibly due to some old adhesions, caused by inflammatory changes.

Another point which Dr. Stockton made, is the continued ill-health of these patients. In the interval they go about doing their work fairly well, that is, in these mild cases he referred to, but there is always a something about them which to the experienced eye shows they are not well. They may not suffer from acute infection, but there is the characteristic odor of these patients, there is the characteristic color, and the lassitude, the malaise, from which they do not entirely recover, and which brands the milder cases.

Now, with regard to the question of resistance in these cases. I recently saw a case in the person of a man at Cortland, who had had repeated attacks of cholecystitis with gallstone formation, and we diagnosticated the case through an obstruction in the common duct. The man had marked jaundice which persisted, and he had at the same time a marked aortic lesion, with very high arterial pressure. Then came up the question of operative intervention. We knew well this man had but little

1. Dr. Curtis's paper was published in the Journal for February, 1904. See page 433.

resistance. On the other hand, we knew he had an obstruction in the common duct which would ultimately kill him. The wisest course seemed to us to recommend and insist upon operation. The operation was performed; the man with arterial sclerosis and advanced aortic lesion withstood the operation and made a complete recovery so far as his cholecystitis and gallstone were concerned. When you have the rigidity, the recurring pains, whether severe or mild to deal with, symptoms in the region of the gallbladder must be taken into consideration, and they need to be correctly interpreted.

The treatment, it seems to me, the more we see of these cases, the more we study the pathology of them, is fast becoming surgical. The results achieved by Reder, by Henschell, and the splendid results we have had in this country, justify operative interference; and I am convinced when we have these cases with persistence of symptoms, with rigidity in the region of the gallbladder, with pains even without gallstones, but with persisting adhesions, the rational treatment is operative. But, also, the treatment which has been so splendidly outlined, the rational treatment by medicine, hygiene, diet, and the like, is always to be considered.

Dr. CURTIS.—I wish to answer the question: all matters of technic I thought I had to exclude because of the short limits of the paper, and I have not time now to discuss the question of drainage or the removal of the gallbladder.

